# Nebulised hypertonic saline (3 %) among children with mild to moderately severe bronchiolitis - a double blind randomized controlled trial

**DOI:** 10.1186/s12887-015-0434-4

**Published:** 2015-09-10

**Authors:** Aayush Khanal, Arun Sharma, Srijana Basnet, Pushpa Raj Sharma, Fakir Chandra Gami

**Affiliations:** Department of Pediatrics, Tribhuvan University Teaching Hospital, Institute of Medicine, Maharajgunj-44600, P.O. Box 1524, Kathmandu, Nepal; Kathmandu Medical College Teaching Hospital, Sinamangal, Kathmandu, Nepal

**Keywords:** Bronchiolitis, Clinical severity score, Epinephrine, Hypertonic saline

## Abstract

**Background:**

To Assess the efficacy of nebulised hypertonic saline (HS) (3 %) among children with mild to moderately severe bronchiolitis.

**Methods:**

Infants aged 6 weeks to 24 months, with a first episode of wheezing and Clinical Severity scores (Arch Dis Child 67:289-93, 1992) between 1 and 8, were enrolled over 4 months duration. Those with severe disease, co-morbidities, prior wheezing, recent bronchodilator and steroid use were excluded. Patients were randomized in a double-blind fashion, to receive two doses of nebulized 3 % HS (Group 1) or 0.9 % normal saline (Group 2) with 1.5 mg of L-Epineprine, delivered 30 min apart. Parents were contacted at 24 h and 7 days. The principal outcome measure was the mean change in clinical severity score at the end of 2 h of observation.

**Results:**

A total of 100 infants (mean age 9.6 months, range 2–23 months; 61 % males) were enrolled. Patients in both groups had mild to moderately severe disease at presentation. On an intention-to-treat basis, the infants in the HS group had a significant reduction (3.57 ± 1.41) in the mean clinical severity score compared to those in the NS group (2.26 ± 1.15); [*p* < 0.001; CI: 0.78–1.82]. More children in the HS group (*n* = 35/50; 70.0 %) were eligible for ER/OPD discharge at the end of 2 h than those in the NS group (*n* = 15/50; 30 %; *p* < 0.001), and less likely to need a hospital re-visit (*n* = 5/50; 10.0 %) in the next 24 h as compared to the NS group (*n* = 15/50, 30.0 %; *p* < 0.001). The treatment was well tolerated, with no adverse effects.

**Conclusions:**

Nebulized 3 % HS is effective, safe and superior to normal saline for outpatient management of infants with mild to moderately severe viral bronchiolitis in improving Clinical Severity Scores, facilitating early Out-Patient Department discharge and preventing hospital re-visits and admissions in the 24 h of presentation.

**Trial registration:**

Clinicaltrials.gov NCTID012766821. Registered on January 12, 2011.

## Background

Bronchiolitis is a common, occasionally severe viral infection of the lower respiratory tract responsible for significant morbidity and mortality in children under two years of age [1]. According to World Health Organization bulletin, an estimated 150 million new cases of clinical pneumonia (principally Pneumonia and Bronchiolitis) occur annually [2]; 11–20 million among them requiring hospital admission. Worldwide, 95 % of all cases occur in developing countries [2]. Epidemiologic data show that RSV accounts for about 65 % of hospitalizations due to Bronchiolitis [3].

Multiple studies [4–7] have documented variation in diagnostic testing, treatment modalities practiced and their outcomes in Bronchiolitis suggesting a lack of consensus for this common disorder. Likewise, despite the frequency of this condition, there is no unanimously accepted evidence driven treatment approach [8, 9]. Besides supplemental oxygen, fluids and supportive care, treatment options include, bronchodilators, epinephrine and corticosteroids [9]. Hypertonic saline (3 %) is a new agent that has been found to be promising in recent studies [10–20]. The proposed mechanism are by improving mucus rheology, reducing airway wall edema and causing sputum induction and cough [12]. A recent meta-analysis [10] also showed a consistent improvement in clinical severity scores and suggested HS may also decrease the length of hospital stay in Bronchiolitis. However, multiple other studies [8, 13, 21–25] have shown equivocal results with little or no clinical benefits with the use of hypertonic saline (3, 6 or 7 %). Similary, there is a paucity of data on comparison of important outcomes like readiness for discharge, need for repeat hospital visits and hospitalization rates, which are important reflectors of morbidity and economic burden [10]. In the paucity of rigorously controlled studies in developing countries using 3 % HS, lack of a consensus regarding management of bronchiolitis in our practice and an opportunity to improve care for this common disorder this study was conducted to assess the therapeutic efficacy of 3 % HS. We tried to study primarily the improvement in CS scores but also looked at parameters like readiness for discharge, need for hospital revisit rates and hospitalization which would reflect the morbidity and financial burden of disease.

## Methods

### Trial design

This study was a prospective, interventional, double-blind randomized controlled trial.

### Ethical clearance

A written informed consent was obtained from the primary caretaker of the patients prior to the enrollment. The study was approved by the Department of Research, Institutional Review Board and Ethics Committee of Tribhuvan University Teaching Hospital.

### Study participants

Subjects were recruited from previously healthy children visiting the Emergency Room (ER) and Out-Patient Department (OPD) of Kanti Children Hospital with the following inclusion criteria:

1. Age between 6 weeks and 2 years
2. First Episode of Wheezing
3. Meets Clinical Definition of Bronchiolitis
4. Clinical severity (CS) scores (Wang et al [26]) between 1 and 9 (Table 1).

**Table 1:** Wang et al. clinical severity score

Variables		Score		
	0	1	2	3
RR	< 30	31–45	46–60	> 60
Wheezing	None	Terminal expiration/only with stethoscope	Entire expiration or audible on exp. without stethoscope	Inspiration and expiration without stethoscope
Retraction	None	Intercostals	Tracheo-sternal	Severe with nasal flaring
General condition	Normal			Irritable, lethargic, poor feeding

Bronchiolitis was clinically defined as per the AAP consensus guidelines [4, 27] as the first episode of acute wheezing in children less than two years of age, starting as a viral upper respiratory infection (coryza, cough or fever).

### Exclusion criteria

1. Any underlying disease (e.g., cystic fibrosis, bronchopulmonary dysplasia and cardiac or renal disease),
2. Prior history of wheezing,
3. Diagnosed case of asthma,
4. Oxygen saturation (SpO2) <85 % on room air,
5. CS score > 9,
6. Progressive respiratory distress requiring mechanical ventilation,
7. Previous treatment with bronchodilators within last 4 h, and
8. Any steroid therapy within 48 h

### Study setting

The study was carried out in the ER, Observation room (OR) and OPD. Recruitment occurred at the peak of bronchiolitis season in between January 15th to April 15th for duration of 4 months.

### Patient assessment

Patient enrollment occurred on weekdays between 08.00 and 17.00 h. The investigator assessed the children for eligibility and assigned a clinical severity (CS) score described by Wang et al. [26] (Table 1). Data were collected using standardized forms to document pertinent history and physical exam. Each children’s weight, temperature, respiratory rate, SpO2 in room air (determined by pulse oximeter, Siemens), heart rate, CS Score and hydration status were recorded. The children were stabilized with antipyretics if necessary (temperature > 38.3*C) and/or nasal suction if the nose was blocked. Supplemental oxygen by face mask was provided to maintain SpO2 > 90 %. Patients determined to be in life threatening condition were immediately managed for same and were not further considered for study.

### Interventions

The study drugs were prepared by a pharmacist, administered by an ER/OPD nurse and compliance with medication administration was assured by the investigator’s direct observation of each nebulization.

All eligible patients were randomly assigned to one of the two groups:

1. Group 1 (*n* = 50) received inhalation of L-Epinephrine 1.5 mg, diluted to 4 ml with 3 % Hypertonic 1 Saline (HS) solution;
2. Group 2 (*n* = 50) received inhalation of L-Epinephrine, 1.5 mg, diluted to 4 ml with 0.9 % Normal Saline solution.

The study drug was administered at 0 and 30 min by a Jet nebulizer using a face mask. The investigator assessed the children’s general condition and recorded the CS score, SpO2, RR and HR prior to each drug administration and at 30, 60 and 120 min after the first nebulization.

**Fig. 1:**
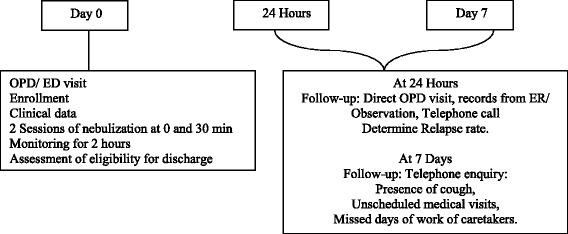
The study design

The study design is shown in Fig. 1.

Adverse events were defined as heart rate > 200, tremor and worsening clinical status. Patients were excluded from the study if the two courses of nebulisation was not delivered, the drug delivery was delayed by 10 min or more (protocol deviation) or if clinical deterioration mandated escalation of therapy and/or support.

The investigator contacted the parents via telephone 24 h after their ED/OPD discharge to determine the need for any unscheduled hospital visit and hospitalization within the next 24 h of OPD/ ER visit: their readmission (relapse) rate. The register at the ER/OR was checked daily for any unscheduled visits by the caretakers. They were also contacted at the end of 1 week in order to record any unscheduled medical visits, missed working days of caregivers and persistence of cough. Patients were labeled as Lost-to-Follow up if there was failure to communicate for 3 consecutive attempts for 2 consecutive days at their 7th and 8th day of initial presentation to the OPD/ER.

### Primary outcome

Compare the mean change in Clinical Severity score among patients with bronchiolitis treated with either L-Epinephrine- 3 % Hypertonic saline or L-Epinephrine- 0.9 % saline.

### Secondary outcomes

- Assess the improvements in SpO2, respiratory rate and heart rate in both intervention groups.
- Compare the discharge readiness and readmission rates in both intervention groups at the end of 2 h of observation and within 24 h following discharge respectively.
- Describe the socioeconomic burden of illness.

### Sample size

Sample size was determined by the following formula:

$$ \mathbf{N} = \left[\left(\mathbf{z}\mathbf{1} + \mathbf{z}\mathbf{2}\right)\mathbf{2}\ \left(\acute{\mathbf{O}} \mathbf{12} + \acute{\mathbf{O}} \mathbf{22}\right)\ \right]\ /\ \left(\overset{\hat{\mkern6mu} }{\mathbf{u}}\mathbf{1}\ \hbox{--}\ \overset{\hat{\mkern6mu} }{\mathbf{u}}\mathbf{2}\right)\mathbf{2}\ . $$

- N: Sample Size
- z1: The confidence level, for *p* value: 0.05, z1: 1.96.
- z2: 0.84 for Power of 80 %
  - 1.28 for Power of 90 %
  - 1.64 for Power of 95 %
- Ó1: Standard Deviation of the Outcome Variable (Clinical Severity Score) in the 1^st^ intervention group (HS)
- Ó2: Standard deviation of the outcome variable (Clinical severity score) in the 2^nd^ intervention group (NS)
- Û 1: Mean change in clinical severity score among 1^st^ intervention group (HS)
- Û 2: Mean change in clinical severity score among 2^nd^ intervention group (NS)
- Allowing a Type1 error of 5 % (α: 0.05), z1 score: 1.96.
- For a Power of 95 %, z2 = 1.64.

The standard deviation of the change in CS score is derived from previous studies [8] and taken as 1.3. We proposed that a difference of 1 point in the CS score between the two intervention groups will be considered clinically significant. To detect this mean difference of 1 unit in the CS score, with a power of 95 %, a sample size of 44 in each intervention group was required. This required a total of 88 patients to be enrolled in the study. Considering the drop out/lost to follow up to be approximately 10 %, 100 patients were enrolled, allowing 50 in each group.

## Randomization

### Sequence generation

A Random Allocation Software [28] generated by computer, identified patients by a triple digit mixed numeric code, was used by the study coordinator to allocate patients to treatment groups, and he was the only person with access to the randomization.

### Type of randomization

Block Randomization method was used to stratify patients into blocks of 10 each, each comprising of 10 patients.

### Allocation concealment

After preparation, the study solutions were labeled with the codes and wrapped in an envelope bearing the respective codes. Study solutions were identical in appearance and odor. Their identity was blinded to all participants, care providers, and investigators and outcome assessor.

### Implementation

Randomization was done by the study coordinator (not involved in the study), who was the only person to have access to the codes. The codes were a mixed 3 digit numeric code. The study solutions prepared by a pharmacist (not involved in the study) were stored in the non-freezer compartment (2–8*C) of the refrigerator and discarded if not used within 72 h of preparation. The investigator assessed the patients and allocated the treatment modalities to each one of them himself.

### Blinding

The study was a Double Blind Randomized Controlled Trial with the investigator, the participants, the nurses who delivered the drug being blinded to the therapeutic option.

### Statistical methods

Statistical analysis was performed using SPSS for Windows, Release 16.0 (SPSS Inc., Chicago, IL). Dichotomous events were analyzed by using the Chi-Square test. Continuous variables were compared by Student *t*-test. Statistical significance was defined as *p*-value < 0.05. This trial has been reported in accordance to the Consolidated Standards of Reporting Trials (CONSORT, 2010) guidelines [29].

## Results and discussion

**Fig. 2:**
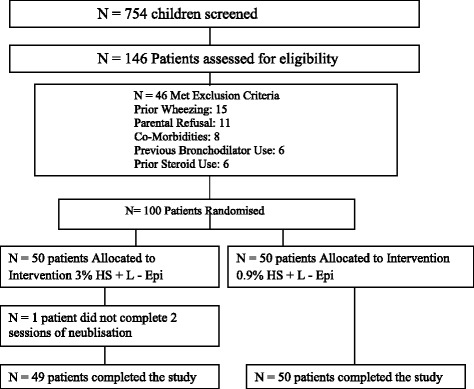
The trial profile

A total of 754 children were screened and 146 previously well children were assessed for eligibility in the study periodas shown in Fig. 2. Forty-Six children were excluded and 99 patients completed the study. Data was analyzed on an intention to treat basis.

No significant differences were noted between the study groups with respect to baseline characteristics (*p* > 0.05) and risk factors for severity as shown in Table 2 and Table 3 respectively. Patients in both groups had moderately severe bronchiolitis with mean CS score above 5. There is gradual improvement in CS score with time in both the groups and the effect seemed to be more pronounced after the second session of nebulisation at 60 min. Patients who received nebulised HS had more significant improvement in the baseline CS scores (Group 1, a change of 3.57 ± 1.41; Group 2, a change of 2.26 ± 1.15; *p* < 0.01) at the end of 2 h of therapy as shown in Table 5. There was also significant difference in the mean change in CS scores, HR, RR and SpO2 between the two groups at the start of treatment and at the end of 2 h of therapy (*p* <0.001) as shown in Table 4 and Table 5. More infants who received HS were eligible for discharge at the end of 2 h as compared to NS [Group 1, *n* = 35(71.0 %); Group 2, *n* = 15(30.0 %); *p* < 0.001] as shown in Table 6.

**Table 2:** Baseline characteristics of 2 groups

Characteristics; mean(SD); (range)	Intervention	Intervention	*p* value
	L-Epi + 3 % HS (*n* = 50)	L-Epi + 0.9 % NS (*n* = 50)	
Age (months), mean (SD); (range)	9.82 (5.06); (2–23)	9.51 (4.28); (3–22)	0.74
Males, n(%)	27 (54.0 %)	21 (42.0 %)	0.40
Duration of illness (days), Mean ± SD	3.43 ± 1.02	3.33 ± 0.96	0.64
Mean (SD) respiratory rate (range)	49.1 (2.5) (46–60)	49.0 (2.0) (46–58)	0.79
Mean (SD) heart rate (range)	148.7 (7.5) (132–168)	149.7 (7.1) (132–166)	0.49
Mean (SD) Spo2 (range)	93.4 (0.8) (90–95)	93.5 (0.7) (90–94)	0.69
Mean (SD) clinical severity score (range)	5.3 (1.6) (3–9)	5.2 (1.1) (3–9)	0.57
Mean temperature (SD) temperature (*C) (range)	37.2 (−17.4) (36.7–38.0)	37.3 (−17.4) (36.6–38.0)	0.82
Urine specific gravity	1016	1022	0.001
ER enrollment, n (%)	2 (4 %)	2 (4 %)	1.00

**Table 3:** Distribution of risk factors proposed to contribute to prolonged disease course and, or severity in both intervention groups

Variables	3 % HS + L-Epi	0.9 % NS + L-Epi	*p*-value
	(*n* = 50)	(*n* = 50)	
Family history of asthma, n (%)	4 (8.0)	5 (10.0)	1.00
Parental smoking, n (%)	30 (60.0)	21 (42.0)	0.05
Exposure to biofuels, n (%)	14 (28.0)	8 (16.0)	0.11
Breastfeeding status, n (%)	48 (96.0)	48 (96.0)	0.75
Prematurity, n (%)	3 (6.0)	2 (4.0)	0.50
Atopic history, n (%)	7 (14.0)	3 (6.0)	0.15

**Table 4:** Mean (± SD) for CS Score, respiratory rate, heart rate and SpO_2_ for patients in each group at 0,30, 60 and 120 min of assessment

	Intervention 0.9 % NS + L-Epi (*n* = 50)	Intervention 3 % HS + L-Epi (*n* = 50)	*p* value	95 % confidence interval lower limits – upper limits (mean)
CS score, n, Mean ± S.D; range				
0 min	5.2 ± 1.1 (3–9)	5.3 ± 1.6; (3–9)	0.57	−0.73; 0.41 (−0.15)
30 min	4.9 ± 1.1; (3–8)	4.3 ± 2.0; (2–8)	0.10	−0.11; 1.21 (0.55)
60 min	3.2 ± 1.0; (1–6)	2.2 ± 1.2; (1–7)	0.001	0.52; 1.43 (0.97)
120 min	2.9 ± 0.8; (1–5)	1.7 ± 0.9; (1–7)	0.001	0.77; 1.52 (1.14)
Respiratory rate, breaths/minute, Mean ± S.D; range				
0 min	49.0 ± 2.0; (46–58)	49.1 ± 2.5; (46–60)	0.79	−1.01;0.81 (−0.12)
30 min	47.4 ± 1.7; (46–54)	46.6 ± 2.1; (42–56)	0.04	0.04; 1.61 (0.81)
60 min	45.3 ± 1.4; (42–50)	43.5 ± 2.0; (40–52)	0.001	1.13; 2.57 (1.85)
120 min	43.6 ± 2.6; (40–48)	40.7 ± 2.0; (38–49)	0.001	1.97; 3.86 (2.91)
Oxygen saturation,%, Mean ± S.D; range				
0 min	93.5 ± 0.7; (90–94)	93.4 ± 0.8 (90–95)	0.69	−0.22; 0.40 (0.09)
30 min	93.8 ± 0.8; (91–96)	94.2 ± 1.2; (90–96)	0.07	−0.79; 0.06 (−0.36)
60 min	94.9 ± 0.9; (92–97)	95.8 ± 1.1; (92–98)	0.001	−1.34; −0.48 (−0.97)
120 min	95.6 ± 1.0; (93–98)	97.0 ± 1.0; (93–99)	0.001	−1.77; −0.91 (−1.14)
Heart rate, beats/minute, Mean ± S.D; range				
0 min	149.7 ± 7.1; (132–166)	148.7 ± 7.5; (132–168)	0.49	−1.94; 3.98 (0.80)
30 min	149.7 ± 7.9; (130–164)	147.1 ± 9.1; (130–164)	0.13	−0.93; 6.04 (2.91)
60 min	145.2 ± 6.4; (130–156)	142.6 ± 7.2; (126–156)	0.06	−0.20; 5.30 (2.55)
120 min	141.8 ± 5.8; (126–154)	138.3 ± 6.6; (124–152)	0.006	1.02; 6.03 (3.53)

**Table 5:** Clinical outcomes in the two intervention groups at the end of 2 h of treatment

Variables (Mean ± SD)	Intervention 3 % HS + L-Epi	Intervention 0.9 % NS+ L-Epi	*p* value	95 % confidence interval (mean)
Change in CS Score	3.57 ± 1.41	2.26 ± 1.15	0.001	0.78; 1.82 (1.30)
Change in HR	10.38 ± 4.54	7.87 ± 3.63	0.003	0.86; 4.16 (2.50)
Change in RR	8.44 ± 2.15	5.40 ± 2.57	0.001	2.08; 3.99 (3.04)
Change in SpO_2_	3.53 ± 0.84	2.12 ± 0.80	0.001	1.07; 1.73 (1.30)

**Table 6:** Secondary outcomes

	Intervention 3 % HS + L-Epi	Intervention 0.9 % + L-Epi	*p* value
Met criteria for ER/OPD discharge after 120 mins, n (%)	35 (70.0 %)	15 (30.0 %)	0.001
Relapse rate, n (%)	5 (10.0 %)	15 (30.0 %)	0.02
Need for intensive care	1 (2.0 %)	1 (2.0 %)	1.00

In addition, in our trial the need for repeat medical visits and hospital admission within the next 24 h of initial hospital visit (Relapse rate) was 20 %, which was significantly lesser in the babies who received HS compared to those who received NS [Group 1, *n* = 5(10.0 %); Group 2, *n* = 15 (30.0 %); *p* < 0.001] as shown in Table 6. During the subsequent week, 78 out of 100 patients (Group 1, *n* = 37; Group 2, *n* = 41) were accessible. As shown in Table 7, a large number of patients in both groups had persistent cough at the end of 1 week (Group 1, *n* = 31; Group 2, *n* = 37; *p* = 0.45). Eighteen patients from HS Group and 23 patients from NS Group had at least 1 unscheduled medical visit within 1 week (*p* = 0.58; overall revisit rates 41/88; 46.5 %) and 3 parents from HS Group and 10 patients from NS Group reported at missing least 1 day of work (*p* = 0.17; total missed days of work 10/77; 12.9 %). No adverse events occurred in either treatment groups. No children were withdrawn from the trial due to side effects or clinical deterioration.

**Table 7:** Burden placed on caretakers due to bronchiolitis

	Intervention 3 % HS + L-Epi	Intervention 0.9 % NS + L-Epi	*p* value
Need for unscheduled medical visits within 1 week, n (%)	18 (36.0 %)	23 (46.0 %)	0.58
Missed days of work	3 (6.0 %)	10 (20.0 %)	0.17
Persistence of cough at the end of 1 week, n (%)	31 (62.0 %)	37 (74.0 %)	0.37

Ours is one of the few studies conducted in South-East Asia with a rigorously controlled design that has not only tried to look at the role of hypertonic saline in improving the CS scores but also studied its impact on early discharge eligibility and hospital revisit rates. In both treatment groups, change in CS was greater than 2 scores suggesting that both treatment combinations were effective. More infants with bronchiolitis were eligible for early discharge and less likely to need any hospital re-visit within the next 24 h (*p* < 0.001). This outcome seems to be particularly relevant in planning resource allocation and staffing in treatment of this common condition.

Similar findings were reported in a recent Cochrane review [10] by Zhang et al., where a total of 560 patients treated with nebulised 3 % saline had a significantly shorter mean length of hospital stay and significantly lower post-inhalation clinical score than the 0.9 % saline group in the first three days of treatment (*p* < 0.001). The effects of improving clinical score were observed in both outpatients and inpatients.

On the Contrary, in another double blind RCT, Wu et al. in 2014 [24] assessed the role of nebulised 3 % HS on change in RDAI scores, admission rates and length of stay in Bronchiolitis. They concluded that HS given to children in the ED decreases hospital admissions but did not produce any significant difference in Respiratory Distress Assessment Instrument score or length of stay as compared to NS.

Likewise, Florin et al. [22] conducted a RCT in ER setting and found that at 1 h after the intervention, the HS group demonstrated significantly less improvement in the median RDAI Score compared with the NS group (*p* < 0.001) and hence concluded that nebulised 3 % Saline was not effective in improving RDAI scores in the ER setting.

Previously, Sarrell et al. [19] had shown that substituting hypertonic saline for normal saline solution (2 ml) in the inhalation mixture for delivering bronchodilator improved clinical scores and decreased hospitalization rates in ambulatory children. In hospitalized children with more severe bronchiolitis, nebulized 4 ml hypertonic saline solution with or without epinephrine was found to be more effective treatment. In our study all infants recovered in both the groups, there was no treatment failure or significant adverse events following nebulisation, as previously reported by Ralston et al. [30].

Our study was well designed to minimize the common bias and limitations associated with research. Sampling bias was addressed by block randomization of patients. Blinding was maintained throughout the study period. A single observer’s assessments nullified the chances of inter-observer variability. Despite these measures, some amount of bias and attrition is inevitable. We could not clarify the additional benefits of supportive care alone in infants with bronchiolitis since we didn’t have a placebo arm. Although we considered important outcomes such as hospital revisit and admission rates, we did not assess the impact of therapy on the length of hospital stay. Our study population consisted mainly of infants who presented earlier, had mostly mild symptoms and experienced significant benefits. We are unsure, if similar benefits could be reproduced in infants with a more severe disease presentation. Although, we had strict inclusion and exclusion criteria, and identified potential risk factors for asthma, we might have included infants with first episode of asthma. RSV testing was unavailable and hence not done. We are also unsure if co-infection worsens the outlook in our infants.

Our study was conducted at the largest tertiary level pediatric referral centre of our country with over 1000 ER visits per month and more than 10,000 OPD visits per month with seasonal variation. Our sampled population characteristics were truly representative of the general population visiting Kanti Children’s Hospital. Strict inclusion and exclusion criteria were used to minimize possible confounding effects of uncharacterized and evolving wheezing phenotypes. A well defined objective, previously validated scoring system was used to assess the clinical response. Our adequate sample size, and double blinded design minimizes the common bias and limitations associated with research. Since our study only consisted of mild to moderate patients with Bronchiolitis, results may need caution while extrapolating to infants with severe disease.

## Conclusion

Nebulised 3 % hypertonic saline in combination with epinephrine was effective in reducing the Clinical Severity scores, meeting the eligibility criteria for OPD/ER discharge and reducing the need for hospital admission among ambulatory children with bronchiolitis. We believe this simple, inexpensive, safe, and effective treatment intervention could minimize the morbidity of Bronchiolitis by generalizing its use in centers caring for pediatric patients. Similar study could be done in multicentric settings with a larger sample size, involving more severely affected patients, and with a placebo control design in order to confirm and extend our results.

## Abbreviations

AAP: American Academy of Pediatrics
CI: 95 % confidence interval
CS: Clinical severity score
ER: Emergency room
HS: Hypertonic saline
OPD: Out-patient department
OR: Observation room
L-Epi: L-Epinephrine
Spo2: Oxygen saturation as measured by pulse oximeter
HR: Heart rate
RR: Respiratory rate
